# Phytoplankton Surveys in the Arctic Fram Strait Demonstrate the Tiny Eukaryotic Alga *Micromonas* and Other Picoprasinophytes Contribute to Deep Sea Export

**DOI:** 10.3390/microorganisms10050961

**Published:** 2022-05-03

**Authors:** Charles Bachy, Lisa Sudek, Change Jae Choi, Charlotte A. Eckmann, Eva-Maria Nöthig, Katja Metfies, Alexandra Z. Worden

**Affiliations:** 1 Monterey Bay Aquarium Research Institute, Moss Landing, Monterey, CA 95039, USA; charles.bachy@gmail.com (C.B.); lsudek@ucsc.edu (L.S.); cjchoi@utexas.edu (C.J.C.); ceckmann@geomar.de (C.A.E.); 2 Ocean EcoSystems Biology Unit, RD3, GEOMAR Helmholtz Centre for Ocean Research Kiel, 24105 Kiel, Germany; 3 Alfred Wegener Institute Helmholtz Centre for Polar and Marine Research, 27570 Bremerhaven, Germany; eva-maria.noethig@awi.de (E.-M.N.); katja.metfies@awi.de (K.M.); 4 Marine Biological Laboratory, Woods Hole, MA 02543, USA

**Keywords:** green algae, phytoplankton, qPCR, sedimentation, carbon flux

## Abstract

Critical questions exist regarding the abundance and, especially, the export of picophytoplankton (≤2 µm diameter) in the Arctic. These organisms can dominate chlorophyll concentrations in Arctic regions, which are subject to rapid change. The picoeukaryotic prasinophyte *Micromonas* grows in polar environments and appears to constitute a large, but variable, proportion of the phytoplankton in these waters. Here, we analyze 81 samples from the upper 100 m of the water column from the Fram Strait collected over multiple years (2009–2015). We also analyze sediment trap samples to examine picophytoplankton contributions to export, using both 18S rRNA gene qPCR and V1-V2 16S rRNA Illumina amplicon sequencing to assess the *Micromonas* abundance within the broader diversity of photosynthetic eukaryotes based on the phylogenetic placement of plastid-derived 16S amplicons. The material sequenced from the sediment traps in July and September 2010 showed that 11.2 ± 12.4% of plastid-derived amplicons are from picoplanktonic prasinophyte algae and other green lineage (Viridiplantae) members. In the traps, *Micromonas* dominated (83.6 ± 21.3%) in terms of the overall relative abundance of Viridiplantae amplicons, specifically the species *Micromonas polaris*. Temporal variations in *Micromonas* abundances quantified by qPCR were also observed, with higher abundances in the late-July traps and deeper traps. In the photic zone samples, four prasinophyte classes were detected in the amplicon data, with *Micromonas* again being the dominant prasinophyte, based on the relative abundance (89.4 ± 8.0%), but with two species (*M. polaris* and *M. commoda*-like) present. The quantitative PCR assessments showed that the photic zone samples with higher *Micromonas* abundances (>1000 gene copies per mL) had significantly lower standing stocks of phosphate and nitrate, and a shallower average depth (20 m) than those with fewer *Micromonas*. This study shows that despite their size, prasinophyte picophytoplankton are exported to the deep sea, and that *Micromonas* is particularly important within this size fraction in Arctic marine ecosystems.

## 1. Introduction

Primary production in high-latitude marine ecosystems is performed by eukaryotic phytoplankton. Among the latter are prasinophytes, a polyphyletic group of green algae containing nano- and pico-phytoplanktonic taxa (≤20 µm and ≤2 µm in cell diameter, respectively; [1]). A number of prasinophytes within the class Mamiellophyceae, including the picoplanktonic *Micromonas* and *Bathycoccus*, have been reported as major contributors to the overall phytoplankton community and biomass in a North Atlantic region influenced by subpolar water masses [2]. Additionally, in the Arctic itself, culturing and PCR-based environmental surveys have exposed one particular *Micromonas* lineage [3], which is also abundant in Arctic sequence datasets [4,5]. This lineage was then observed in Antarctic waters based on metagenomic analyses [6], with a possible connection between poles via deep-ocean currents, and has now been described as a distinct species, named *Micromonas polaris* [7]. *M. polaris* represents one of the seven or more lineages comprising the genus *Micromonas* [6]. Collectively, these lineages are considered as sentinels for ocean change, in part because of their differing sensitivities to water temperature [8,9]. Changes in communities and the importance of *Micromonas* have already been documented. For example, as early as 2009, increased abundances were observed in the Canadian Arctic that correlated with increased water temperature and decreased salinity, while, in parallel, larger algae, such as diatoms, decreased [10].

Despite the apparent abundance and predicted rise of *Micromonas* among phytoplankton community members in warming Arctic Oceans, its contribution to vertical export is largely unknown. A variety of studies have characterized large particles (>100 µm) and their export to the deep ocean (e.g., [11,12]), extending to particles in, e.g., the 11–64-micrometer range, as well as their importance [13]. However, for many years, it was widely believed that small organisms, such as picophytoplankton, that predominate in the open ocean, do not contribute to carbon export from the surface to deeper layers of the water column due to their slow sinking rates and possible rapid remineralization in the microbial loop [11,14]. Actual sediment trap data are more limiting for small particles because it is difficult to observe the entire size range of particles by using a single methodology, and smaller cells cannot be identified visually [13]. However, both observational and modeling studies have now demonstrated that small plankton, including picoplankton, contribute to export from the photic zone through a variety of processes [15,16]. Picophytoplankton, including prasinophytes, have been detected in particles in 4000-meter sediment traps in the subtropical North Pacific Gyre [17]. *M. polaris* has also been detected in North Atlantic Deep Water (at 3000 m) and is thought to be present there due to thermohaline circulation processes [6]. At high latitudes, 0.2–20-micrometer-sized particles thought to be surface-derived have been reported at depths of 1000 m using in situ optical scattering instruments in the Norwegian Sea, at ca. 70° North latitude [18]. These results provide evidence that particles in this size range contribute to long-term carbon sequestration but cannot be used to identify particle origins—whether they represent small cells, and, if so, which types, or potentially fragments of detrital material. Overall, these studies highlight the need for methods that identify the types of small cell that contribute to export.

The first goal of this study was to examine vertical microbial connectivity by assessing the composition of surface and exported phytoplankton communities in polar waters. Specifically, we studied samples in the photic zone from a transect across the Fram Strait, the only deep-water connection between the Arctic and Atlantic Oceans. In addition to the transect, we analyzed samples from the Long-Term Ecological Research Site (LTER) HAUSGARTEN, established in the year 2000, which is in the eastern part of the Fram Strait [19]. To determine the taxa that contribute to export, we used amplicon sequencing to examine phytoplankton communities in deep sediments traps (200, 1250 and ~2500 m), with the deepest depth being near/at the seafloor, as well as samples from the above sunlit zone. Our analyses focused on the summer season, which has been shown to be the period with the highest documented carbon flux to the sediment in the Fram Strait [20]. The observation that *Micromonas* was omni-present in the transect allowed us to compare its abundance between the seasonally ice-covered Western Fram Strait and the mainly ice-free Eastern Fram Strait. We also assessed the contributions of *Micromonas* along a longitudinal transect in the Fram Strait over a 6-year period (2009–2015) using more quantitative methods (qPCR) [21]. The second goal of this study was to elucidate the extent to which *Micromonas* is exported from the upper water column to the seafloor. Therefore, we also quantified the contributions of *Micromonas* in the top 100 m of the water column in summer 2010, and in the deepest sediment trap samples. The results provide the first insights into the prasinophyte species that are exported to the deep sea, and into how *Micromonas* contributes to primary production and carbon export in the rapidly changing Arctic environment.

## 2. Materials and Methods

### 2.1. Sample Collection

For sampling of sinking particles, material was collected by modified automatic Kiel sediment traps with a sampling area of 0.5 m^2^ and coupled with 20 liquid-tight collector cups and one lander trap (Lander), with a sampling area of 0.25 m^2^ [22,23]. Here, we present results from the deepest sediment traps (200, 1250, and ~2500 m below sea surface, named Oben, Mitte, and Unten, respectively) and a lander trap (2.5 m above the seafloor, at a 2380-meter depth) at the central station (HG-IV) of the LTER observatory HAUSGARTEN at 79°00.41′ N, 04°19.83′ E (bottom depth ~2550 m). Collector cups remained open for ~2 weeks (between 10–16 days) until closing and arrival of the next collector cup. Trap samples were collected through summer and autumn 2010 (Supplementary Table S1). Detailed information on the sediment trap collection, preservation, and sample preparation for DNA isolation can be found in [24] and https://doi.pangaea.de/10.1594/PANGAEA.845616 and https://doi.pangaea.de/10.1594/PANGAEA.855472 (both accessible as of 23 April 2022). Briefly, collection cups of 400 mL were filled with filtered seawater, adjusted to a salinity of 40 and spiked with mercury chloride at a final concentration of 0.14% to preserve samples during the deployment and after recovery. Samples were then stored at 4 °C until the time of analysis; large zooplankton (>0.5 mm) were removed under a dissecting microscope. Next, for the purpose of this study and flux calculations (see below), we used a split of the original sediment trap sample (ranging from 1/512 to 1/128, Supplementary Table S1) that was filtered onto a 0.2 µm Isopore GTTP membrane filter (Millipore, Burlington, MA, USA), washed, and stored at −20 °C.

Water-column samples from above the region of sediment traps and beyond were collected using 12-liter Niskin bottles mounted on a CTD rosette from two different stations of HAUSGARTEN at depths of 5 (HG-I), 25, and 100 m (HG-IV) in mid-July 2010. To assess Fram Strait *Micromonas* abundances, we used samples collected during depth profiles (5 to 100 m) at some HAUSGARTEN sites (S-III, HG-I, HG-IV, HGN-IV, and HG-IX) in the same mid-July 2010 period and, subsequently, from different depths between the surface (~5 m) and 55 m along a longitudinal transect in the Fram Strait over a 6-year period (2009–2015). The depths chosen for sampling reflect the surface and the depth of chlorophyll (Chl) maximum when present. Two-liter subsamples were taken in PVC bottles from the Niskins. Planktonic cells were collected by sequential filtration of one water sample through three different mesh sizes (10, 3, and 0.4 µm) on 45-millimeter-diameter Isopore Membrane Filters at 200 mbar using a Millipore Sterifil filtration system (Millipore, Burlington, MA, USA). The Sterivex membranes were then stored at −20 °C until further processing. 

CTD data from depth profiles collected during the POLARSTERN cruise ARK-XXV/2 [25] and other environmental metadata are accessible on PANGAEA. Sampling map was produced using Ocean Data View v5.2.1 (available online at: https://odv.awi.de accessible as of on 23 April 2022).

### 2.2. DNA Isolation

For sediment traps, genomic DNA was isolated from the samples with the E.Z.N.A Plant DNA Kit (Omega Bio-Tek Inc., Norcross, GA, USA) [24]. The original protocol was modified with an additional washing step in order to remove residual mercury chloride, which could have inhibited PCR amplification. This step used “SPW wash buffer” and was only needed for sediment trap samples (as water-column samples did not use mercury chloride).

For seawater samples, genomic DNA was extracted from the individual filters using the E.Z.N.A TM SP Plant DNA Kit manufacturer’s dry specimen protocol. The extracts from the different filter sizes were pooled and stored at −20 °C until analysis.

### 2.3. High-Throughput Sequencing and Analysis

Amplicons were generated using the 16S rRNA V1-V2 primers 27F_ill (5′-TCGTCGGCAGCGTCAGATGTGTATAAGAGACAGagrgttygatymtggctcag-3) and 338R_ill (5′-GTCTCGTGGGCTCGGAGATGTGTATAAGAGACAGgcwgccwcccgtaggwgt-3′; capital letters represent Illumina linker sequences on the 27F/338R primer pair) as in [26] and purification was performed using a MinElute kit (Qiagen, Hilden, Germany). Amplicon products were sequenced using the Illumina Miseq platform. Primer sequences were cropped out using CutAdapt software [27] to remove a fixed number of bases (-u parameter) matching the 27 F (20 bp) and 338 RPL (18 bp) primer lengths. Trimmed fastq files were quality-filtered, dereplicated, and merged with dada2 R package, version 1.2 [28]. Potential chimeras were removed de novo using the removeBimeraDenovo command. Post-quality-control 16S rRNA amplicons were classified using a modified version of PhyloAssigner [29], as described in [2]. They were first placed on the global 16S rRNA gene reference tree using maximum-likelihood methods [25] for characterization as either plastids/cyanobacteria or heterotrophic bacteria; those with best node placements to the former were subsequently placed using the same phylogenetic methods on a cyanobacterial and plastid 16S rRNA gene reference tree [30]. Amplicons assigned to the viridiplants and stramenopiles in this second classification step were then respectively placed on Viridiplantae [2] and Stramenopila [31] reference trees for final taxonomic assignment, again using maximum-likelihood methods for phylogenetic placement. Along with the Viridiplantae tree, we also developed an additional stramenopile alignment to further identify diatom sequences. We used reference sequences assigned as stramenopile plastids in PR2 v4.14.0 [32]. Redundancy was removed from the PR2 database first, with clustering using CD-HIT v.4.6 [33] at 99% nucleotide identity. This final processed PR2 database and resulting diatom reference tree contained a total of 832 sequences. Diatom amplicons from our study were then mapped onto the PR2 reference tree using EPA-ng, following the same procedures as in [26] to taxonomically identify amplicons.

### 2.4. Statistical Analyses

Statistical analyses included Spearman correlations, Mann–Whitney analyses, and t-tests, depending on the data type and data distribution. Statistical tests were performed in SigmaStat V 14, as provided within SigmaPlot.

### 2.5. Quantitative PCR

To quantify *Micromonas* we used a *Micromonas*-specific TaqMan primer-probe set targeting the 18S rRNA gene [34]. Quantitative PCR (qPCR), including inhibition tests and analyses, were performed using methods described previously [34] for three temporal sediment trap samplings (i.e., a total of 21 samples) and five seawater profiles (i.e., a total of 25 samples). In addition, 56 seawater samples collected along the East–West Fram Strait transect at different depths (5–55 m) between 2009 and 2015 were analyzed. Collectively, this resulted in a total of 102 samples analyzed by qPCR.

The 18S rRNA gene copies per mL in seawater samples were determined based on cycle threshold (*C*T) values fitted on linear regression of *C*s versus copy numbers (in log scale) of the standard curve, also taking into account sample volume filtered through the 0.4-micrometer filters (between 500 mL and 2 L, Supplementary Table S2), elution volume, dilution, and template volume. For 18S rRNA gene copy fluxes (per m^2^ and per day) in sediment traps, we also took into consideration the split of the original trap sample, the surface area of the trap, and sampling period in days.

## 3. Results

**Figure 1:**
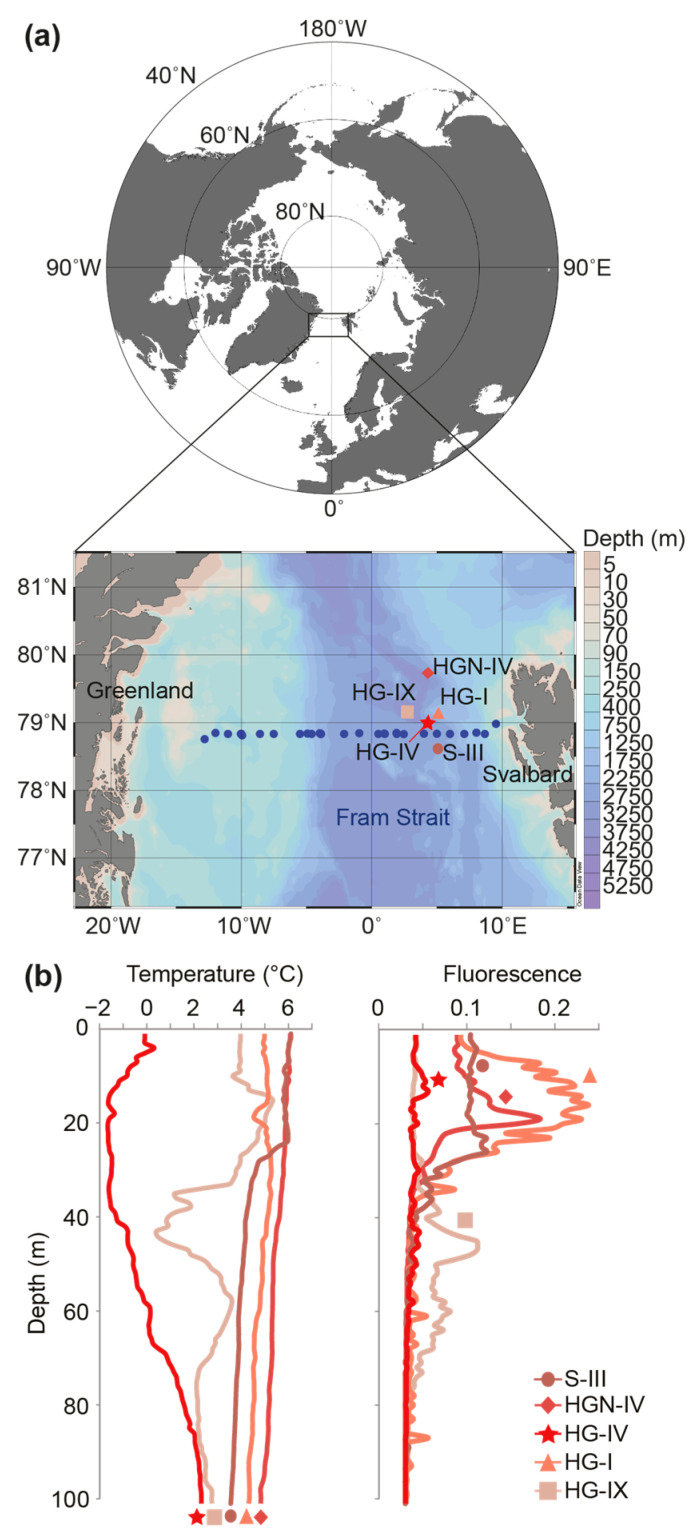
Location and characteristics of stations sampled. (**a**) Fram Strait map with sampling sites of the Long-Term Ecological Research (LTER) observatory, HAUSGARTEN, and of the Svalbard-Greenland transect (in blue). The moored sediment traps and lander were located at HG-IV, indicated by the star. (**b**) Temperature and in vivo chlorophyll *a* fluorescence over depth profiles at the five LTER HAUSGARTEN stations sampled in July 2010. Symbols along the bottom connect to station symbols in (**a**).

The water column profiles at the HAUSGARTEN LTER stations sampled in July 2010 showed no thermal stratification at HG-I and HGN-IV (Figure 1). A similar temperature range (4–6 °C) was found at station S-III, which did exhibit a modest stratification gradient at a depth of around 25–30 m. The characteristics of these three stations are typical of Atlantic water that originates from the subpolar and subtropical North Atlantic gyres that are transported northward within the West Spitsbergen Current. By contrast, the water column at the station HG-IV displayed a colder water mass, with temperatures of 0 °C and below, which are classically observed on the most westerly part of the strait, which transports cold, fresh water of Arctic origin [35]. The station HG-IX showed a somewhat intermediate profile, with water at 4 °C above 30 m, a drop to a minimum temperature at around 40 m (0 °C), and at depths below varying between 1 and 3.5 °C. This temperature profile is likely to represent a transitional state between different water masses. Sub-surface Chl maxima were observed between 10 and 25 m for the depth profiles typical of the Atlantic waters (Figure 1b), but were not observed at HG-IV or HG-IX.

**Figure 2:**
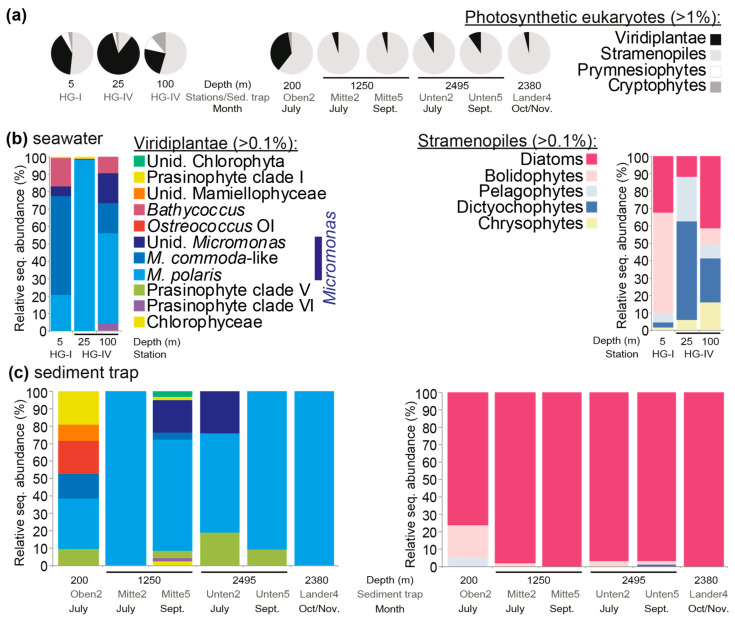
Eukaryotic phytoplankton composition in summer 2010 at the HAUSGARTEN LTER. (**a**) Relative abundance of V1-V2 16S rRNA gene amplicons from major groups of plastid-containing organisms. Upper water column data come from stations HG-I and HG-IV, based on sampling on 12 July 2010, and data from moored sediment traps come from sample collections in summer and autumn 2010. Only groups detected at >1% at the respective sites/traps are shown. (**b**) Relative abundance of different Viridiplantae groups (green algae), largely prasinophyte algae, and also of stramenopile groups, relative to the total for each group in the respective surface-layer-water samples. (**c**) The same as (**b**) except that the data are from trap samples. In all cases, amplicons were taxonomically assigned using Phyloassigner following the methodology and alignments used in Choi et al. (2020); the color coding in (**c**) is the same as in (**b**).

Phytoplankton community composition was evaluated at the study sites using DNA samples from different regions of the water column, the photic zone, and below the photic zone (using moored and lander traps). The amplicon primers used in this study amplified the 16S rRNA gene V1-V2 hypervariable regions, thus capturing most of the microbial diversity, including bacteria, cyanobacteria, and eukaryotic plastids. The V1-V2 16S rRNA gene sequencing of three summer seawater (July 2010, HG-I, HG-IV) and six sediment trap (summer 2010) samples resulted in 504,700 total amplicons post-quality-control (56,078 ± 13,318 amplicons per sample). Four major phytoplankton groups were detected at relative abundances >1%: viridiplants (green algae, primarily prasinophytes), stramenopiles, prymnesiophytes (i.e., haptophytes), and cryptophytes. During the summer season, green algae contributed 24–84% of the total eukaryotic phytoplankton amplicons at the surface (Figure 2a). The rest of the eukaryotic phytoplankton was a mixture of stramenopiles, prymnesiophytes, and cryptophytes, among which the stramenopiles had the highest relative abundances (Figure 2a). Among the green algae, three classes were detected. The dominant green algal genus was *Micromonas*. *M. polaris* was more relatively abundant at the HG-IV station, with a subsurface maximum at 25 m. At the HG-I station, *M. commoda*-like (Clade C sensu [6,36,37]) represented 56% of the Viridiplantae amplicon abundance, while other *Micromonas* species were seen, as well as *Bathycoccus*, which was also true for the 100-meter HG-IV sample (Figure 2b). For these same stations, bolidophyte dominated among the stramenopile amplicons at HG-I (5 m), while dictyochophytes and diatoms dominated the stramenopile amplicons at depths of 25 and 100 m (HG-IV), respectively.

Sediment trap samples showed lower relative abundances of Viridiplantae amplicons than observed in surface samples. It should be highlighted that very little is known about the efficacy of mercury chloride in preserving small cells with minimal cell wall structure, such as *Micromonas*. Specifically, the material sequenced from the six sediment traps on two dates showed that 87.7 ± 13.9% of plastid-derived amplicons were from stramenopiles and that 11.2 ± 12.4% were from green algae (Figure 2a). Among the latter, four prasinophyte classes were detected, with sequences attributed to *Micromonas* dominating in the sediment traps (83.6 ± 21.3% of the Viridiplantae amplicons), predominantly *M. polaris* (Figure 2c). At Oben2 (with a collection depth of 200 m), several taxa that were only observed in trace amounts or not detected in the surface water were recovered. These were the prasinophyte class V (i.e., Pycnococcaceae), *Ostreococcus* clade OI, and unidentified Mamiellophyceae. *M. polaris* dominated Viridiplantae amplicons in this sample, and in all other trap samples as well. The dominant stramenopile amplicons in sediment traps appeared to be mostly from diatoms (Figure 2c), particularly an unidentified species of the genus *Chaetoceros* (Supplementary Table S3). Dictyochophytes and pelagophytes, as well as bolidophytes, were low in relative abundance in all trap samples except Oben2 (200 m, July).

**Figure 3:**
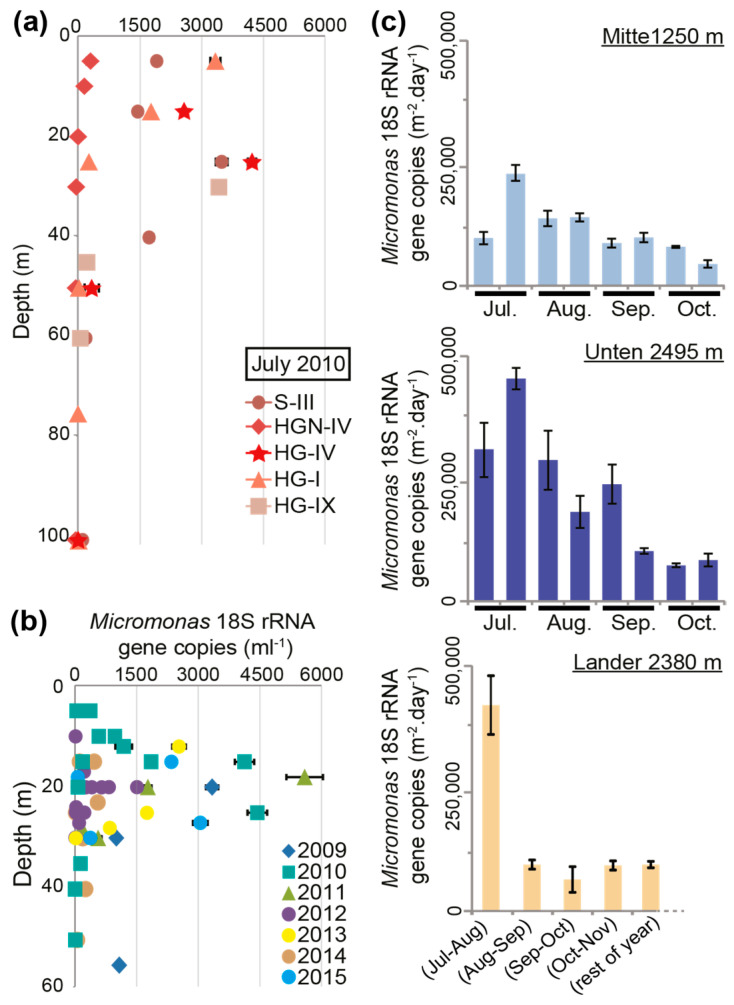
A multi-year view of *Micromonas* abundances in the Fram Strait. (**a**) *Micromonas* (minimum) 18S rRNA gene copies per mL by qPCR (which, due to losses during extraction, etc., could only capture minimum values) of filtered seawater samples from depth profiles at the five LTER HAUSGARTEN stations sampled by seawater filtration in the month of July, 2010. DNA surface samples are missing above 15 m at HG-IV and above 30 m at HG-IX. (**b**) *Micromonas* (minimum) 18S rRNA gene copies per mL from stations along the Svalbard–Greenland transect over a seven-year period that were collected at the subsurface chlorophyll maximum, as defined based on the in vivo chlorophyll *a* maximum. (**c**) Deposition (or detectable remainders) of *Micromonas* in the moored long-term sediment traps and the bottom lander positioned at HAUSGARTEN site HG-IV in 2010. Error bars reflect the standard deviation of technically triplicated qPCR measurements.

In order to gain a better sense of the potential abundances of the dominant picophytoplankton genus, *Micromonas*, we employed *Micromonas*-specific Taqman qPCR primer-probes on the HAUSGARTEN samples (Figure 3a). Total cell concentrations in the photic zone varied considerably. In the unstratified profiles at HGN-IV and HG-I (Figure 1b), the peak abundances were dramatically different (260 ± 38 and 3343 ± 121 *Micromonas* 18S rRNA gene copies per mL, respectively), but both occurred in the surface samples, even though for HG-I, the in vivo chlorophyll *a* fluorescence showed a wide region of high subsurface chlorophyll (Figure 3a). Combined with the amplicon sequencing, this indicated that the in vivo fluorescence profile was shaped by large fractions of both stramenopiles and prasinophytes, but that the former may have had increasing importance deeper in the water column. At stratified S-III (Figure 1b), a prominent peak of 3495 ± 142 *Micromonas* 18S rRNA gene copies per mL was seen at 25 m, corresponding to the position of the fluorescence maximum, thermocline, and nutricline. The *Micromonas* concentrations at S-III remained relatively high (1457 to 1919 gene copies per mL) to the surface, akin to patterns seen in the in vivo chlorophyll *a* fluorescence profile. Similarly high abundances were observed at depths between 25 and 30 m at HG-IV and HG-IX (>3425 gene copies per mL). In the coldest profile studied (HG-IV), *Micromonas* showed peak abundance in the colder region of the photic zone (Figure 1b), specifically 4232 ± 101 gene copies per mL at 25 m (−1.7 °C), although at 15 m concentrations were also relatively high (2584 ± 40 gene copies per mL; −1.3 °C). The peak in this polar-influenced station corresponded to a depth where *Micromonas* formed 83% (12,565 out of 15,049) of all the plastid-derived amplicons.

We performed a number of analyses to examine possible trends in the HAUSGARTEN data. We did not find statistically significant correlations with environmental parameters in the HAUSGARTEN samples from the surface to a depth of 50 m (with tests including as well as excluding depths below 50 m because they were potentially outside the photic zone). However, we found that the samples with abundances >1000 *Micromonas* 18S gene copies per mL had significantly (*p* < 0.001) lower standing stock phosphate than those with lower mean abundances, with 2614 ± 991 gene copies per mL and 0.24 ± 0.11 µmol L^−1^, respectively, versus 77 ± 129 gene copies per mL and 0.41 ± 0.20 µmol L^−1^. Nitrate (3.0 ± 2.6 vs. 7.1 ± 3.1 µmol L^−1^) and depth (17 ± 9 m vs. 32 ± 18 m) exhibited the same relationship as seen for phosphate, but silicate and temperature did not. The lower *Micromonas* abundances seen at higher nitrate, phosphate, and deeper photic zone depths may connect directly to depth and, potentially, to reduced light availability, rather than nutrient concentrations per se. Alternatively, these relationships may arise from diminished competitive advantage of picophytoplankton (relative to larger taxa) once nutrient concentrations increased. Hence, the competitive advantages of the larger phytoplankton, such as diatoms, which formed a larger fraction of the plastid-derived amplicon sequences deeper in the surface layer, could also have influenced these observations (Figure 2a,b).

Prior experiments on cultures have shown that the two detected *Micromonas* species (*M. commoda* and *M. polaris*) have different temperature optima [3,8]. *M. polaris* CCMP2099 exhibits growth at 0 °C, but has not been tested at lower temperatures [3]. Clade A, B, and C isolates (sensu Simmons et al., 2015; *M. commoda* and *M. commoda*-like) grow over broader ranges of temperature than *M. polaris*, and separate into warmer- and colder-adapted isolates. The strain RCC1697 (*M. commoda*-like), isolated in the North Sea, where the seasonal low is 6 °C, has been shown to survive at 4 °C and to grow at temperatures of up to 25 °C (albeit slowly) [8], whereas *M. polaris* dies above 12 °C [3]. Here, the two stations with the highest *Micromonas* gene copy abundances detected also had amplicon data. In combining these data types, we found that the Atlantic influenced HG-I, with a temperature of 6 °C and 3343 ± 121 *Micromonas* gene copies per mL, exhibited the highest relative abundance of *M. commoda*-like cells. Likewise, the polar-water-influenced station HG-IV also showed high abundances, specifically 4232 ± 101 gene copies per mL, with water temperatures below 0 °C (−1.7 °C), and nearly all its prasinophyte amplicons were from *M. polaris.*

We also evaluated multi-year surface and sub-surface samples (from depths between 5 to 55 m) along the East–West transect between Svalbard and Greenland (Figure 1a and Figure 3b). QPCR was performed on samples selected for having the maximum in vivo chlorophyll *a* fluorescence value relative to the other depths in the sampled water column. In general, samples with the highest *Micromonas* concentrations appeared at depths between 15 and 25 m depth and >1000 18S rRNA gene copies per mL were observed in all the years except for 2014. Furthermore, we found that the *Micromonas* concentrations of >1000 18S rRNA gene copies per mL (range 1005 to 5594) typically came from samples with higher temperatures (n = 14, median 5.25 °C, *p* < 0.05) than those with lower concentrations (n = 42, median 2.59 °C; range 2 to 972). Nevertheless, several samples in the pool with >1000 gene copies per mL came from low-temperature samples (−1.2 to −1.7 °C)—again likely reflecting the presence of *M. polaris*, rather than that of *M. commoda*-like strains. Our primer-probe sets did not distinguish between *M. polaris* and *M. commoda*-like species, potentially explaining why, overall, the correlation-based analyses of abundance versus temperature did not suggest coherent relationships. The peak *Micromonas* abundance observed was at station HGN-IV, at 18 m (5.45 °C), with 5594 ± 444 18S rRNA gene copies per mL.

We also quantified *Micromonas* 18S rRNA gene copies in the sediment trap and lander samples using qPCR. The data exhibited temporal variation, with higher *Micromonas* fluxes in summer traps (July–August) at 1250 m (“Mitte”), 2380 m (“Lander”), and 2495 m (“Unten”). Abundances peaked within each temporal monitoring during the second half of July at 1250 and 2495 m (236,823 ± 15,498 and 451,764 ± 20,732 gene copies per m^2^ per day, respectively) and, during the 30 days of sampling between mid-July and mid-August, they peaked at 2380 m (418,763 ± 61,052 gene copies per m^2^ per day) (Figure 3c). 

## 4. Discussion

The western part of the North Atlantic Ocean near Baffin Bay and the Labrador Sea (Canadian Arctic) and the eastern part, at the Fram Strait and the Greenland Sea, exhibit the “most intense absorption of anthropogenic carbon globally” [20], and the biological carbon pump plays a key role. In the Canadian Arctic, the abundance of picophytoplankton in the photic zone, specifically *Micromonas*, has been shown to be increasing due to the influence of climate change at the same time as larger phytoplankton decline [10]. Observations from the Fram Strait’s photic zone have led to the proposal that small pico- and nanoplankton may be replacing diatoms during summer in this ecosystem as well [38]. However, unlike relatively large diatoms, which are thought to sink directly, picophytoplankton have classically been portrayed as non-sinking particles. This raises questions about which taxa are exported and how export might be affected by climate change, with Arctic regions undergoing the most rapid environmental change. The collective of *Micromonas* species has been proposed as an effective tracer of change due to differing thermal tolerances and distributions [7,8,9,39]. With regard to environmental changes, the Fram Strait captures an important cross-section of interacting water masses, from polar waters to Atlantic waters as well as coastal regions subject to the influences of melting ice and climate-related perturbations (Figure 1a). The surface waters in the eastern location of HAUSGARTEN are characterized as having less ice coverage and higher water temperatures than the western part of the Fram Strait [40]. Export can be examined in underlying waters using moored sediment traps extending down to the seafloor in the HAUSGARTEN region [25,41]. Our studies build on the rich background of knowledge on primary production and export in this region, and the rare opportunity to gain insights into vertical particle flux patterns for tiny algal cells.

Plankton-microscopy-based studies are longstanding in the Fram Strait and at the HAUSGARTEN LTER, in both the ice-free photic zone and in sediment traps [38,42,43,44]. The majority of sediment trap studies to date have focused on large phytoplankton, which can be identified to the genus level, and sometimes beyond, by microscopy. For example, the diatom *Chaetoceros* has been observed by microscopy in the upper part of the Fram Strait’s photic zone, usually from the beginning of the growing season under the form of resting spores [45]. This diatom genus has large cells bearing long spines and is in the nano- or micro-plankton size spectrum. We observed *Chaetoceros* in the photic zone via amplicon sequencing and, in addition to other diatoms, a collection of more diverse stramenopiles, such as dictyochophytes and bolidophytes (Figure 2), some of which can be difficult to distinguish by microscopy. In the traps, we found that the stramenopiles were almost exclusively dominated by *Chaetoceros* amplicons, while amplicon proportions of pico-eukaryotic bolidophytes were very low. In comparison to prasinophytes, diatom frustules and structures that facilitate easier identification may also lead to better preservation than some of the dominant pico-prasinophytes, such as *Micromonas* and *Ostreococcus*, which are soft-bodied and have no visible cell walls. Additionally, *Chaetoceros*, with its large size, is more likely to be subject to a known export process (sinking) than these prasinophyte algae. Thus, few studies have addressed eukaryotic phytoplankton that are less readily identifiable, such as picoplankton, and their contributions to carbon flux.

Molecular sequencing has dramatically altered the information that is available on Fram Strait and HAUSGARTEN phytoplankton communities—revealing a diversity of picoplanktonic genera that were not previously known to reside there. Indeed, pyrosequencing Tag studies (18S V4) revealed that *Micromonas* is common in the Fram Strait and that other prasinophytes reside there as well [40,46]. As we move towards the interpretation of our findings from the water column and sediment trap samples, it is important to pause and discuss the factors that differentiate our approaches from those used in prior reports, as well as possible caveats. Here, we characterized community compositions using plastid-derived 16S rRNA V1-V2 sequences, not V4 or V9 18S rRNA amplicons. Some variable regions cannot resolve different taxa well, and there are large variations in how well they perform, as shown for 18S V4 and V9 when applied to prasinophytes [47]. Additionally, plastid 16S rRNA gene copies are more constrained than 18S rRNA gene copies, with the latter varying widely across protistan and other eukaryotic organisms. Therefore, plastid 16S amplicons have been described as more accurately reflecting the contributions of phytoplankton to the total photosynthetic community in pelagic environments than 18S amplicons [31,48]. These factors limit the efficacy of direct comparison of 18S and 16S amplicon taxonomic relative abundance assignments. 

DNA preservation and extraction from cells may also shape amplicon and qPCR data. For example, there is the possibility that the fixative used for sediment trap samples (i.e., mercury chloride) may have greater or lesser success with different cell types. Similarly, the efficacy of traps in capturing different cell types and particle sizes likely varies [24,49]. Furthermore, sediment trap samples reflect the communities captured from a large area over a time-period of multiple days, while the water-column samples are only snapshots of small volumes on a particular day. With respect to the overall quantitation, the qPCR numbers reported herein represent *Micromonas* cells per mL of about half that value, depending on the number of 18S rRNA gene copies present in the *Micromonas* genomes (currently between two and three, depending on the genome assembly studied) [9,50]. At the same time, we know that there are DNA losses all along the extraction procedure, and, hence, that values should be taken to reflect a minimum cell concentration. We found that the overall total *Micromonas* 18S rRNA gene copies per mL detected in the Fram Strait were roughly equivalent to those for *Micromonas* in the eastern North Pacific (ENP) [51], although in the ENP, other species dominate, such as *Micromonas pusilla*. Conversion of our qPCR data to *Micromonas* cell concentrations also rendered similar quantities to those reported based on epifluoresence microscopy in the central Arctic Ocean’s photic zone during the late spring and summer growing season [52].

Our plastid amplicon (16S V1-V2) analyses, which used phylogenetic placement methods, allowed us to clearly delineate the species present. We detected the amplicon sequences from prasinophyte clade I (i.e., Pyramimonadales), prasinophyte clade II (i.e., *Bathycoccus*, *Ostreococcus* clade OI, *M. commoda*-like, *M. polaris*, and other, unidentified, lineages), prasinophyte clade V (i.e., Pycnococcaceae), and clade VI (i.e., *Prasinoderma* considered as a member of a recently erected Viridiplantae phylum [53]). Initially, *M. pusilla* was reported as being present in this region [3,36], with later recognition [3,7] that those present were in fact *M. polaris*, alongside a potential member of the *Micromonas* Clade C [5,40]. Several pyrosequencing-based studies have reported *Micromonas* (often misidentified as *M. pusilla*) in the Fram Strait, including an unidentified *Micromonas* at sites HG-I, S-III, and HG-IV, with high relative amplicon abundances. Some of these studies acknowledge that the sequences were possibly more similar to *M. polaris* than to *M. pusilla*, and that a species related to isolates within the Clade A, B, C lineage was present (Sensu [6]) [40,46]. Evidence exists for the presence of *M. polaris* in surface waters of various other regions of the Arctic Ocean at high relative amplicon abundances, including locations throughout the central Arctic [54] and a fjord in the polar Atlantic [55]. Additionally, a study combining terminal-restriction fragment-length polymorphism analysis and the cloning/sequencing of the 18S rRNA gene showed *M. polaris* in the Beaufort Sea [56]. Herein, the V1-V2 region of the 16S rRNA gene resolved *Micromonas* clades and demonstrated that, primarily, *M. polaris* and *M. commoda*-like are present, as well as the presence of a still-unidentified *Micromonas* at lower relative abundances.

Our results emphasize the importance of recognizing the evolutionary distances between *M. commoda* (Clade A.II, sensu [6]), represented by the genome-sequenced strain RCC299 [50], and *M. commoda*-like species (Clade C.I), which are clearly discriminated in multi-marker genes and some 18S rRNA and 16S rRNA gene analyses [6,36]. Interestingly, the *M. commoda*-like lineage (i.e., Clade C.I, sensu [6]) isolates largely come from cold-water environments, such as CCMP1195 and RCC1697, which were isolated from the Gulf of Maine (in November) and in the North Sea, respectively. In the latter, the average sea-surface temperature is 10 °C, but temperatures as low as 6 °C are observed, which are akin to those seen at the sites where *M. commoda*-like cells were abundant. The seeming coexistence of *Micromonas* species in the Fram Strait’s photic zone can be refined further, since it follows the type of patterning reported for species of the picoprasinophyte *Ostreococcus*. Specifically, similar phenomena are seen for *Ostreococcus* species in the frontal regions of the Kuroshio Current in the North Pacific [57] and in the western North Atlantic [2]. This patterning connects to physical dynamics in that coexistence and even enhancement are seen in frontal regions (where the intermingling of water masses occurs) and dominances seemingly arise in water masses with more coherent origins and characteristics. Additionally, the eddying recirculation observed in this area of the Fram Strait could transport species adapted to cold waters towards warmer waters, and vice versa [20].

Studies on the Fram Strait’s photic zone based on 454 pyrosequencing have proposed that *Micromonas* are favored in (relatively) warmer conditions [40,46]. It is important to recognize that some trends in relative abundances may also result from the influences of different dynamics of templates from other organisms on relative Tag/amplicon abundances. Our quantitative data allowed us to deeply characterize the distributions with regard to the true abundance of this important genus. We observed that there was a higher average temperature overall for samples with higher *Micromonas* abundances, and that the species in the warmer, more Atlantic-influenced stations were different from those in the cold waters. However, *M. polaris* is found at equivalently high abundances in waters <−1.0 °C, which were encountered less frequently in the heavily sampled HAUSGARTEN region (reflecting temperature/location biases in our samples) but are more common in the colder, western part of the Fram Strait. Hence, the impact of climate change on the water dynamics in this region will certainly affect the species distribution of different *Micromonas* and, potentially, export. For now, their cellular abundances in the photic zone can be observed at equally high levels over the broader system, although with differences in species distributions.

*Micromonas* and other prasinophytes have been reported in shallow sediment traps (110 m) based on 18S rRNA gene amplicon pyrosequencing in the Kara Sea. This Arctic Sea does not have the same Atlantic influences as the Fram Strait/Greenland Sea and has considerable ice cover from October to June. A 10-month trap deployment initiated in September, when the surface water temperature was between 0.2 to −0.4 °C, showed a high relative abundance of *M. polaris* throughout the September–June sampling period [58]. The sampling was performed monthly (remotely, through the rotation of the trap collection vessels) under the ice, with no additional surface monitoring due to its frozen state. The Fram Strait also has ice cover, the extent of which has declined significantly since 1979 but, typically, is at its greatest in April. Several possibilities might underpin the massive dominance of *M. polaris* among the prasinophyte amplicons in sediment traps. For example, it raises the possibility that *M. polaris* has alternate forms or cell states that are transported to the deep efficiently (i.e., a faster sinking rate) or are preserved more effectively. Alternatively, it may simply reflect the temporal or physical offset of the surface samples from the communities captured in the traps—since, for example, the communities detected in the sediment traps can be ‘disconnected’ for days or months from those in the photic zone at the collection time. This potential offset should be monitored by sampling surface waters (when ice-free), physical flow at multiple depths, and sediment traps, in order to relate exported material with the surface productivity and the communities from which it is derived. For example, there is another current around Spitsbergen, which is very cold, that could be an alternative source of advected *M. polaris* to the more western sea-ice area. Overall, the polar trap data from near the seafloor in the Fram Strait and below the photic zone in the Kara Sea show similarities in the massive dominance of *M. polaris* over other *Micromonas* types, other prasinophytes, and indeed any other type of photosynthetic pico-eukaryote.

The flux of the *Micromonas*, based on the quantitative analysis of the trap data, exhibited temporal variation, with peaks in the middle of the summer (second part of July; Figure 3c). Strong seasonality in eukaryotic microbial community composition and in the export related to meltwater-derived stratification, is reported for the Fram Strait [20,59], and other means of export exist through mesoscale features [15] in the eastern North Atlantic. Likewise, the influence of seasonality on both biological composition and carbon export appears to be very strong at more temperate and subtropical sites, where sediment traps have been monitored using molecular approaches in the North Pacific gyre (HOT [10]) or by comparing two seasons in the North Atlantic gyre (BATS; [60]). The temporal changes we observed in *Micromonas* abundance in the traps could reflect (i) changes in *Micromonas* productivity in the surface ocean, (ii) a faster export related to aggregation (i.e., phytodetritus and fecal aggregates or pellets), (iii) the influence of ice-melt on stratification, or (iv) potential changes in physical transport related to water currents, mesoscale, and submesoscale processes. Moreover, the results from the sediment trap deployments in 2007 and 2008 in the Fram Strait show that a significant part of the collected material comes from the sides, through lateral advection [42]. As communities shift in connection to climate change, it becomes increasingly important to establish temporal patterns and decipher the mechanisms transporting picophytoplankton and other taxa to depths where they prevail, and possible alterations in the future.

*M. polaris* was discovered in the Canadian Arctic [3,61] and, as mentioned above, was shown to increase in abundance in association with climate-induced changes to the ecosystem [10]. *M. polaris* is also present in the Antarctic Ocean, according to amplicon, metagenomic, and qPCR studies [6,26]. It has been hypothesized that the presence of *M. polaris* in both Arctic and Antarctic waters could be explained by a possible connection between poles via the thermohaline processes responsible for the global ocean conveyor belt, specifically North Atlantic Deep Water (NADW) [6]. Our results provide the first data supporting the proposed mechanism underlying its presence in the deep oceanic currents involved in global transport, which was first inferred from the presence of *M. polaris* in mid-latitude NADW waters (at 3000 m) and in Labrador Sea surface waters, where water sinks to form NADW [6]. Although the equivalent value is not currently known for the Labrador Sea due to the lack of sediment trap data, the results herein demonstrate exactly the needed export in the Fram Strait, from which deep waters ultimately join into the sources of the NADW that flows to Antarctica and other locations. These findings further establish how transport from the Arctic to Antarctica occurs for algae that can only tolerate cold temperatures, such as *M. polaris* [3,8], whose route must circumvent surface waters [6]. Altogether, a new generation of studies is needed that involves the time-series assessment of presence and abundance in water columns and sediment traps in the context of physical water masses, as well as their intermingling and movement. These studies will be needed in order to truly characterize pico-eukaryotic contributions to carbon export from surface to deep waters and the seafloor.

Von Appen et al. (2021) emphasize that “As Arctic sea-ice melts, the biological carbon pump changes, impacting global climate and other critical ocean attributes (e.g., biodiversity).” The Fram Strait encapsulates these types of change, with the modification in sea ice extent that has been developing for decades (i.e., the decrease in sea ice thickness and increase in sea ice extent) [62]. Because we collected data from various years in the warmer eastern part of the Fram Strait and along the Svalbard–Greenland transect, our quantitative assessments serve as an important initial set of baseline data for monitoring picophytoplanktonic community changes, both in the photic zone and in the material exported to the seafloor. Our studies clearly suggest that picoplanktonic prasinophytes, with a predominance of *M. polaris* but including other genera and species as well, are exported to the deep sea. Combined with the Kara Sea study [58] and other relative amplicon abundance studies showing *M. polaris* as the dominant pico-eukaryote during summer [3,4,54,55,56], our findings indicate that pico-prasinophytes contribute to the biological carbon pump in other Arctic export regions. However, while we demonstrate the export of picoplanktonic cells, with DNA-based identification down to the species level, the results do not address the explicit mechanisms through which these small particles were exported to greater depths. Documented shifts in polar phytoplankton community size structure point to increases in small photosynthetic eukaryotes in the Canadian Arctic [10]. Moreover, *Micromonas’* high relative abundances have now been established across multiple Arctic sites, alongside the high absolute numbers and demonstration of its export presented herein. Hence, our results underscore the urgent need to better understand explicit export mechanisms of small cells and the overall contribution of picophytoplankton to the biological carbon pump.

## Data Availability

Detailed information on the sediment trap collection, preservation, and sample preparation for DNA isolation can be found at https://doi.pangaea.de/10.1594/PANGAEA.845616 and https://doi.pangaea.de/10.1594/PANGAEA.855472. Metadata on samples were extracted from PANGAEA websites. The amplicon sequencing data set generated for this study can be found in GenBank, under accession numbers SAMN27660762 to SAMN27660770. The final processed, dereplicated stramenopile sequences from PR2 and the resulting diatom reference tree are deposited in GitHub and can be accessed via github.com/cbachy/Arctic-Micromonas.

## Supplementary Materials

The following supporting information can be downloaded at: https://www.mdpi.com/article/10.3390/microorganisms10050961/s1. Table S1: Moored sediment trap samples. Table S2: Seawater samples. Table S3: List of dominant diatom amplicons. Table S3: List of diatom Amplicon Sequence Variants (ASVs) representing >1% of stramenopile reads per sample with their corresponding sequences and taxonomic assignment using phylogenetic placement.
